# Supplementary material for: CACNA1C as a Prognostic Biomarker and Therapeutic Target in High-Grade Serous Ovarian Cancer: Clinical Validation and Molecular Dynamics of Nifedipine Blockade
Source: bioRxiv. 2026 Apr 22:2026.04.19.719516. Preprint. [Version 1] doi: 10.64898/2026.04.19.719516 (PMC13131657; doi:10.64898/2026.04.19.719516)
Supplement: 1 [file NIHPP2026.04.19.719516V1-supplement-1.pdf]

bioRxiv preprint doi: https://doi.org/10.64898/2026.04.19.719516; this version posted April 22, 2026. The copyright holder for this preprint (which was not certified by peer review) is the author/funder, who has granted bioRxiv a license to display the preprint in perpetuity. It is made available under aCC-BY 4.0 International license.

Supplemental Information (SI): Figures and Table:

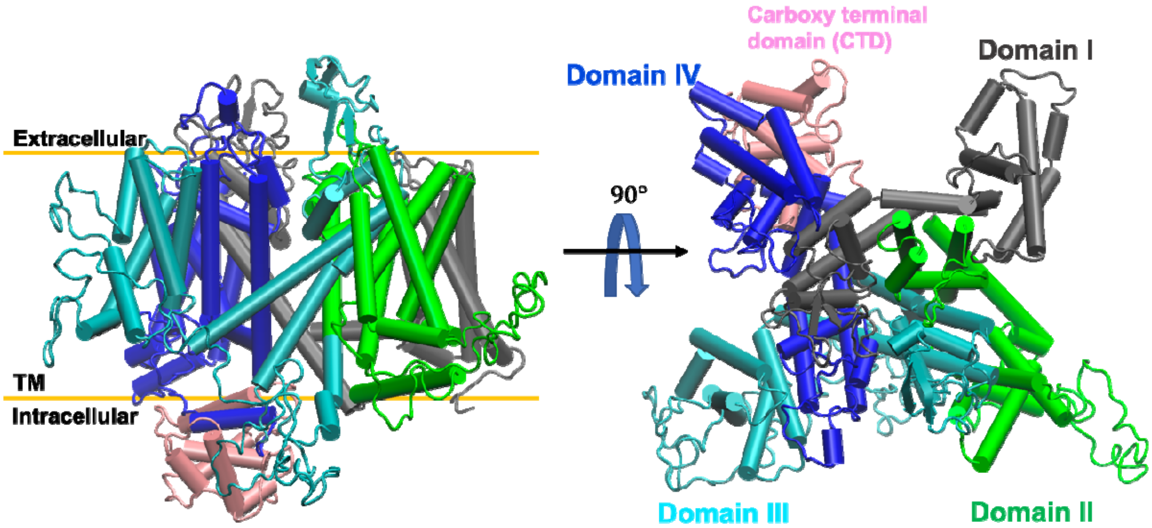

SI Figure 1. Structure of the constructed hCaV1.2 channel. Structure of the hCaV1.2 channel generated by homology modeling from lateral view (left) and rotate 90° from extracellular view (right). Scheme of cartoon representation for hCaV1.2 structure with four domains and carboxy terminal domains are indicated; domain I in gray, domain II in green, domain III in cyan, and domain IV in blue. Each domain is composed of six transmembrane helices (S1-S6), where the first four, S1-S4, formed the voltage-sensing domain (VSD) while the other two, S5-S6, formed the pore domain (PD). The extracellular segment that connects helices S5 and S6 formed two re-entrant short helices denominated P1 and P2.

bioRxiv preprint doi: https://doi.org/10.64898/2026.04.19.719516; this version posted April 22, 2026. The copyright holder for this preprint (which was not certified by peer review) is the author/funder, who has granted bioRxiv a license to display the preprint in perpetuity. It is made available under aCC-BY 4.0 International license.

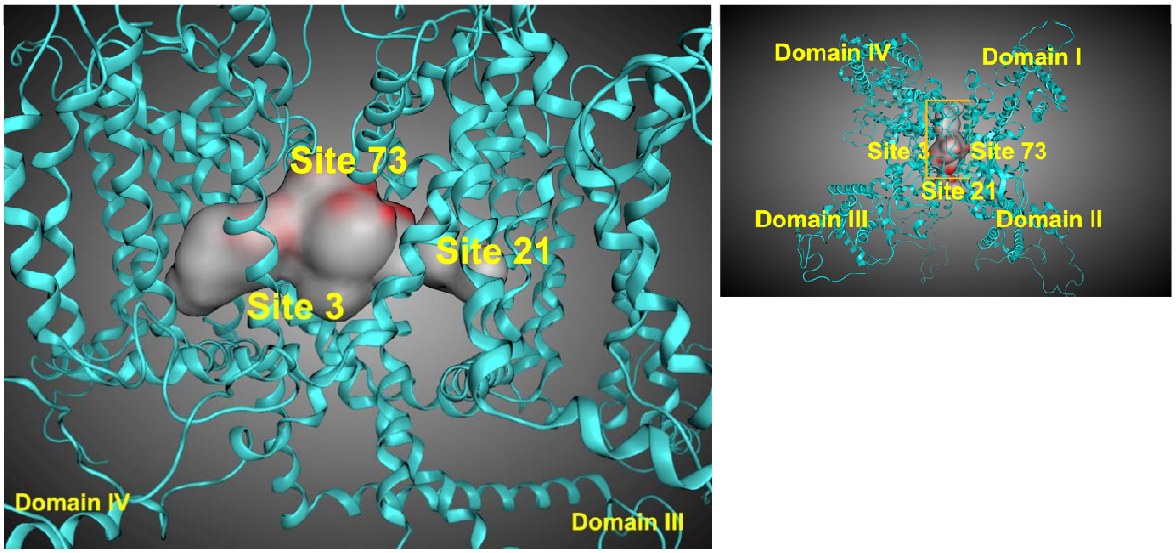

SI Figure 2. Ligand binding sites in the structure of the constructed hCaV1.2 channel. Three sites were found within the pore of the hCaV1.2 channel. Site numbers were indicated by the order found in the site finder tool.

SI Table 1: Binding modes with the relative scorings of NIFE.

Binding mode	Score (kcal/mol) (Nifedipine)
Site 3 mode 1	-6.89
Site 3 mode 2	-6.82
Site 3 mode 3	-6.76
Site 3 mode 4	-6.75
Site 3 mode 5	-6.67
Site 21 mode 1	-7.30
Site 21 mode 2	-7.22
Site 21 mode 3	-7.17
Site 21 mode 4	-7.10
Site 21 mode 5	-7.00
Site 73 mode 1	-7.72
Site 73 mode 2	-7.29
Site 73 mode 3	-7.24
Site 73 mode 4	-7.20
Site 73 mode 5	-7.10

bioRxiv preprint doi: https://doi.org/10.64898/2026.04.19.719516; this version posted April 22, 2026. The copyright holder for this preprint (which was not certified by peer review) is the author/funder, who has granted bioRxiv a license to display the preprint in perpetuity. It is made available under aCC-BY 4.0 International license.

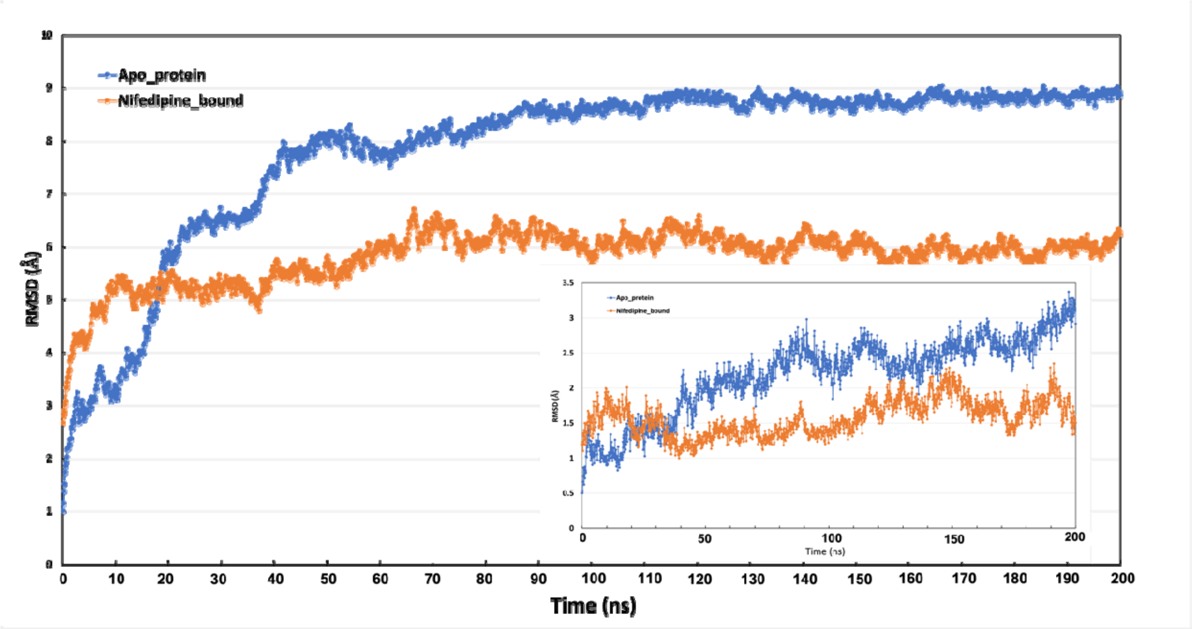

SI Figure 3. Comparison of the RMSD over 200 ns MD simulations between the Apoprotein and NIFE bound structures. Inlet is the RMSDs of ligand bound pocket.

bioRxiv preprint doi: https://doi.org/10.64898/2026.04.19.719516; this version posted April 22, 2026. The copyright holder for this preprint (which was not certified by peer review) is the author/funder, who has granted bioRxiv a license to display the preprint in perpetuity. It is made available under aCC-BY 4.0 International license.

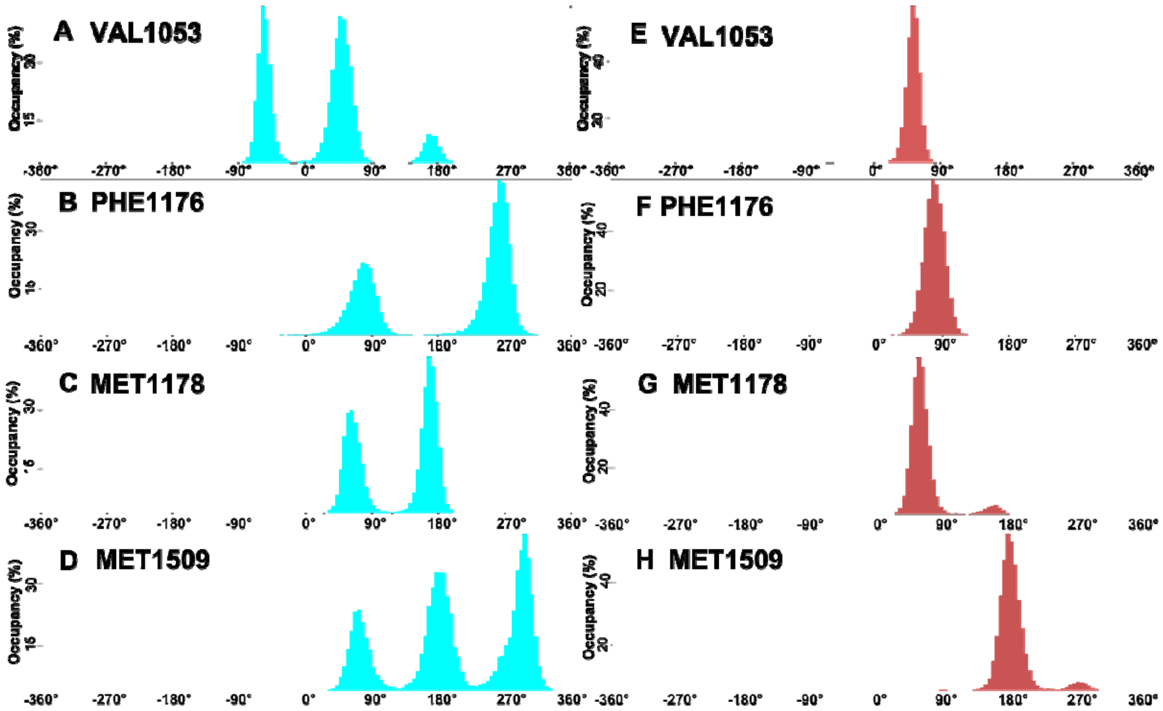

SI Figure 4. Clustering conformations of residues surrounding the ligand binding pocket. Each histogram can represent one rotameric state. (A)/(E) (Val1053), (B)/(F) (Phe1176), C/G (Met1178), D/H (Met1509). Left panel is for apoprotein structure and right panel is for NIFE bound structure.
